# YOLOv5s-SA: Light-Weighted and Improved YOLOv5s for Sperm Detection

**DOI:** 10.3390/diagnostics13061100

**Published:** 2023-03-14

**Authors:** Ronghua Zhu, Yansong Cui, Jianming Huang, Enyu Hou, Jiayu Zhao, Zhilin Zhou, Hao Li

**Affiliations:** 1 School of Electronic Engineering, Beijing University of Posts and Telecommunications, Beijing 100876, China; 2 SAS Medical Technology (Beijing) Co., Ltd., Changping District, Beijing 102200, China

**Keywords:** sperm detection, depthwise separable convolution, YOLOv5, attention mechanism

## Abstract

Sperm detection performance is particularly critical for sperm motility tracking. However, there are a large number of non-sperm objects, sperm occlusion and poorly detailed texture features in semen images, which directly affect the accuracy of sperm detection. To solve the problem of false detection and missed detection in sperm detection, a multi-sperm target detection model, Yolov5s-SA, with an SA attention mechanism is proposed based on the YOLOv5s algorithm. Firstly, a depthwise, separable convolution structure is used to replace the partial convolution of the backbone network, which can ensure stable precision and reduce the number of model parameters. Secondly, a new multi-scale feature fusion module is designed to enhance the perception of feature information to supplement the positional information and high-resolution of the deep feature map. Finally, the SA attention mechanism is integrated into the neck network before the output of the feature map to enhance the correlation between the feature map channels and improve the fine-grained feature fusion ability of YOLOv5s. Experimental results show that compared with various YOLO algorithms, the proposed algorithm improves the detection accuracy and speed to a certain extent. Compared with the YOLOv3, YOLOv3-spp, YOLOv5s and YOLOv5m models, the average accuracy increases by 18.1%, 15.2%, 6.9% and 1.9%, respectively. It can effectively reduce the missed detection of occluded sperm and achieve lightweight and efficient multi-sperm target detection.

## 1. Introduction

Infertility is a common health problem, affecting 15% of couples worldwide [1]. Overall, 30% of infertility cases are related to male infertility [2]. A fundamental challenge in the diagnosis and treatment of infertility lies in the rapid and accurate analysis of semen samples provided by the laboratory. Sperm concentration, morphology [3,4] and DNA fragments are important indicators for semen analysis [5]. In addition, another important indicator affecting male fertility is sperm motility [6,7,8,9], and high-quality detection methods are the key to accurately assessing sperm motility.

Sperm detection is a complex and challenging process. In semen micro videos, sperm cells are usually unstained, colorless and transparent. Color, size and texture information were not effective in sperm detection and are basically ineffective in sperm detection based on color, size and texture information. On the other hand, sperm cells have highly similar morphology, which greatly reduces the feasibility of detection algorithms to extract information from the morphology [10,11,12]. At the same time, semen samples are doped with a lot of epithelial cells or other impurities, which put forward higher requirements for accurate detection of sperm in video frames.

As far as we know, the initial sperm detection methods relied on manual marking by clinical experts using various medical counting devices [13,14,15]. However, manual evaluation is highly subjective, labor-intensive and subject to differences within and among technicians, who lack rigorous and objective data support. To compensate for this, the computer-assisted semen analysis (CASA) [16,17,18] system has been introduced to provide a more accurate, objective and standardized sperm assessment for this field of diagnosis. However, due to the strict parameter definition and many uncertainties of the algorithm used, the methods of sperm detection still vary greatly. New ways are needed to automate and standardize the sperm detection process. In the literature [19], Jati G et al. detected the position of sperm based on the Kalman filter algorithm and inferred the motion trajectory of each sperm by using the Hungarian method. However, the problem of sperm detection in the low contrast region of an image sequence has not been solved. Hasikin et al. [20] proposed a region-based adaptive threshold segmentation technique that used the intensity distribution to classify image pixels, group and separately process pixels, generate multiple thresholds according to the classification and grouping, and realize spermatozoon segmentation in low-contrast areas. When motile sperm collide with impurities with similar contrast, it is easy to lose the sperm target, resulting in a certain percentage of sperm-tracking loss. Alameri et al. [21] adopted an improved Gaussian mixture model to detect sperm and automatically update, including new sperm that re-entered the field of vision. When the number of swimming sperm is large enough, the calculation speed of the algorithm is greatly reduced. Qi et al. [22] detected moving objects through background modeling, and at the same time, realized the classification and counting of sperm with progressive motion, non-progressive motion and non-motility. For detection of small targets, Alabdulla et al. [23] proposed a new method to optimize non-uniform illumination images, and adopted an improved branch-bound algorithm for sperm location trajectory allocation, which had a low error rate in sperm detection and tracking. Somasundaram et al. [24] proposed a FRCNN based on an elliptic scan detection algorithm (ESA) for human sperm motility analysis, improving the accuracy of computer-assisted semen analysis. In recent years, with the wide application of deep learning, experts and researchers at home and abroad continue to deepen and expand the research on small-target detection technology and have made some achievements. Yet, only a handful of algorithms use deep learning to detect sperm cells. Prabaharan et al. [25] used image morphological processing to reduce noise, segmented sperm images by the E-Ostu threshold method, and then used a convolutional neural network to detect abnormal regions. The method achieved 98.99% accuracy and was able to effectively detect abnormal sperm morphology. Abbasi et al. [26] applied a novel combination of deep transfer learning and multitasking learning to sperm detection. The literature [27] uses classical CNN to detect and segment sperm cells. Compared with traditional algorithms, one of the advantages of this work is that it can process stained semen images. In 2022, Qixian et al. [28] established a new dataset consisting of 1207 sperm cell images from more than 20 male infertility patients. They proposed an efficient deep learning algorithm integrating extended convolution into the U-Net network structure for automatic segmentation of the human sperm head. However, the introduction of extended convolution is prone to a grid effect, which will lose the continuity of feature information. The object detection algorithm based on deep learning uses a large number of datasets to train the model, which has a stronger generalization ability and is more suitable for complex and changeable scenes.

In this paper, the proposed method is based on the deep convolution neural network of YOLOv5s [29] architecture for sperm detection. The main contributions of this study are as follows:

- We propose a novel YOLOv5s algorithm and introduce Shuffle Attention (SA) mechanism [30] to enhance the model’s attention to small target sperm.
- We propose to replace partial convolution in the backbone network with depthwise separable convolution (DWConv) [31] to improve the convergence speed of the model.
- The proposed method can effectively process semen images with low quality and a complex background, improve the detection performance of sperm and provide the feasibility of accurate detection using human or other animal sperm cells.

The rest of the paper is organized as follows. Section 2 describes the creation of the sperm dataset and the detailed structure of the YOLOv5s-SA detection model. Experimental comparisons of the state-of-the-art detection methods are performed in Section 3. Finally, Section 4 states our conclusions and discusses future work.

## 2. Materials and Methods

### 2.1. Date Set

The quality of the dataset is critical to the outcome of deep learning. Currently, the only open-source dataset for sperm motility prediction is VISEM [32], but this dataset does not provide detailed data consistent with deep learning detection. Therefore, we built a dataset. In the R&D department of SAS Medical Technology (Beijing) Co., LTD. (Beijing, China), there were microscopic videos of sperm from 10 different men with unstained samples. To capture sperm movement, we used a phase contrast microscope with a total magnification of 100 times. Videos range in length from 10 s to 130 s, are shot at 30 fps and have a resolution of 1280 by 960 pixels. The average number of sperm per frame is 274.6.

In this study, datasets were constructed from video samples of 10 different males’ sperm, and the first 100 frames were captured from the first 6 male semen video samples, 80% of which were used for the training dataset and the rest for the validation dataset. The training set had 480 images and 133,921 sperms. The validation set consisted of 32,768 sperms. In addition, 30 video frames were randomly captured from the remaining 4 videos and used as a test dataset. The test set had 120 pictures and contained 33,194 sperm cells. Table 1 illustrates the dataset proportions. The training set and the test set are strictly independent. Although there are only 10 videos in the data, there are a significant number of annotated objects.

### 2.2. YOLOv5s-SA Architecture

**Figure 1:**
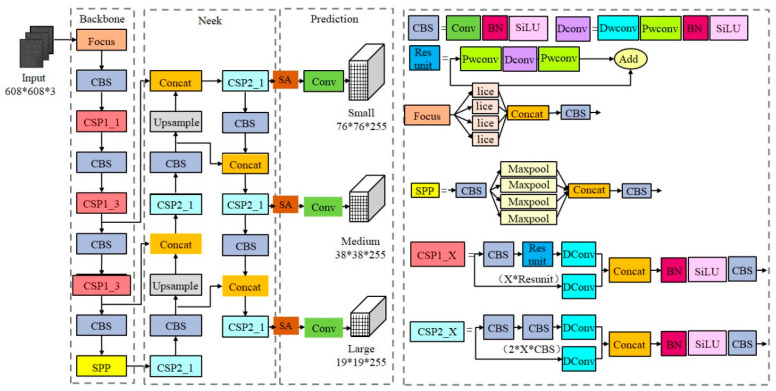
Structure of YOLOv5s-SA model.

The structure of the YOLOv5s-SA target detection network is shown in Figure 1, which consists of the backbone feature extraction network, neck network and head target prediction network. (1) The backbone network is mainly composed of the focus, CSP module and spatial pyramid pooling (SPP) [33] module. In order to reduce the number of parameters in the model, partial convolution in the backbone network is replaced by DWConv [31]. (2) The feature pyramid network (FPN) [34] and path aggregation network (PAN) [35] are used in the neck to realize multi-scale feature fusion. In order to further enhance deep and shallow semantic feature fusion and suppress redundant background noise, we introduced an SA attention mechanism [30] to enhance the model’s attention to small target sperm. (3) The head prediction network integrates features of the acquired enhanced feature maps and introduces EIoU instead of CIoU [36] to calculate the bounding box regression losses and obtain the prediction results.

#### 2.2.1. Lightweight Backbone Feature Extraction Module

**Figure 2:**
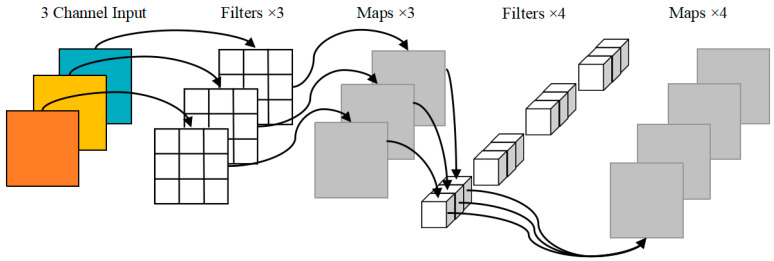
DWConv model.

Skip connections between different layers of the original YOLOv5 backbone network can alleviate the gradient disappearance caused by the deepening of the network. However, due to the excessive number of CSP structures, the texture details are easily lost. We designed a lightweight backbone network to reduce the weight parameters and computation amount of the model. DWConv of MobileNet [31] replaces the partial convolution of the backbone network, which can guarantee the sperm detection accuracy is not affected while the number of parameters and calculation of the model are not increased. The specific structure of DWConv is shown in Figure 2, which is a combination of depthwise convolution and pointwise convolution, with a low parameter quantity and calculation cost. In addition, the selection of the activation function in the convolution operation has a certain impact on the detection performance inspired by the literature [37]. We replace the LeakyReLU [38] activation function in the convolution layer with SiLU [39] with good generalization ability, whose expression is defined as follows:

(1) f(x)=x⋅sigmoid(βx)

where β is a constant or trainable parameter. SiLU has the characteristics of non-monotonic, lower limit, no upper limit and smooth, resulting in a stronger regularization effect. The specific structure of the lightweight backbone is shown in Figure 1.

#### 2.2.2. Multi-Scale Feature Fusion Enhancement Module

The original YOLOv5s network extracted features from the shallow layer to the deep layer. Shallow layer features have higher resolution and rich geometric information, but lack detailed feature information and have a small receptive field. On the contrary, the deep feature layer has high semantic information and a strong receptive field due to multiple convolution, but at the cost of losing part of the resolution and location information. After the analysis of the self-built dataset, there is only one target category to be detected in the sample, and each sperm to be identified in the sample is basically a small target. The deeper features are fused with the shallower features. The dimensions of the feature maps’ output from different network layers are unified by an up-sampling or down-sampling operation. The concat operation is used to fuse multi-scale feature information. The network model has a high resolution, strong receptive field and rich semantic information.

Let the input channels of the branches at both ends of the concat operation be $X_{1},X_{2},\ldots ,X_{c}$ and $Y_{1},Y_{2},\ldots ,Y_{c}$. Then, the output of concat is shown in Formula (2).

(2) $$Z_{concat}=\sum_{i=1}^{c}X_{i}*K_{i}+\sum_{i=1}^{c}Y_{i}*K_{i+c}$$

where ∗ denotes the convolution operation. As can be seen from the above formula, the concat layer only realizes the concatenation of two or more feature maps on the channel dimensions. That is, the features of the image itself are increased, but no attention is paid to the relationships between each feature in the channel. Cross-scale feature fusion easy causes an aliasing effect, resulting in semantic information attenuation. Inspired by the SA module [30], the spatial attention and channel attention mechanisms can be efficiently combined in feature fusion networks. After inserting the SA module into the three output channels of the neck network, we introduced group convolution to reduce the amount of computation and assigned corresponding weights to each group to enhance the perception of details.

**Figure 3:**
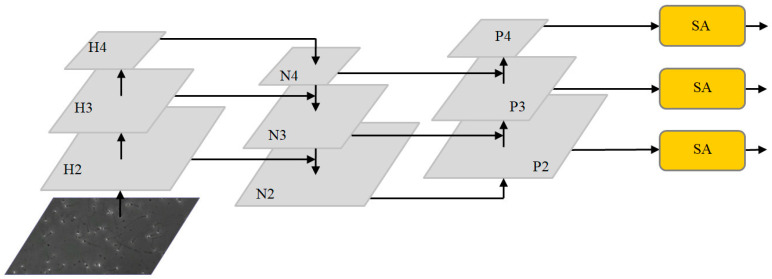
The optimized multi-scale fusion structure.

Feature fusion algorithms based on channel attention and spatial attention are applied to the neck network. The optimized multi-scale feature fusion network is shown in Figure 3. After extracting image features from the original image through the backbone network, three feature maps with different depths are obtained: H2, H3 and H4. The feature map is then input to the neck network. First, the FPN structure conveys strong semantic features from the top-down, incorporating deep feature information into the shallow layer. After concat and the up-sampling of the deep feature map H4 and middle layer feature H3, N3 is generated by the convolution operation. Feature map N3 is then fused with shallow feature map H2 by up-sampling to generate the enhanced feature map N2. Because FPN is limited by unidirectional feature fusion, shallow feature information is not fully utilized. The PAN structure further carries out feature fusion on the feature layer and integrates the shallow feature information into the deep layer. N2 is down-sampled and fused with feature layer N3 to obtain the enhanced feature figure P3. P3 is down-sampled and fused with feature layer N4 to obtain the enhanced feature figure P4. Finally, feature maps P2, P3 and P4, combined with the efficient SA modules, enable the neck network to accurately learn the correlation of feature maps between channels, alleviate background information and noise interference from non-sperm cells and enhance the feature extraction effect of target sperm.

### 2.3. Parameter Setting and Experimental Environment

In addition to the neural network architecture, network parameters are also crucial for training speed and accuracy. We set the size of video frames sent into network training as 608 × 608 pixels, batch_size as 8, epoch as 1000 and initial learning rate as 0.01. We set the momentum factor to 0.9 and the weight attenuation parameter to 0.0005. Mosaic data enhancement is adopted to increase the diversity of the training samples and improve the generalization ability of the network.

The training and testing environment of the YOLOv5s-SA algorithm is as follows. The GPU is NVIDIA GeForce GTX 1080ti, and the processor is Intel(R) Core(TM) i7-6700. The operating system is Windows 10, and pytorch 1.9.1 + CUDA 10.2 is selected as the deep learning framework.

## 3. Experiments

### 3.1. Evaluation Metrics

In order to evaluate the performance of the algorithm, the precision (P), recall (R), average precision (AP) and $F_{1}$ score ($F_{1}$) are used as four evaluation indexes of the model detection accuracy. Their definitions are as follows:

(3) $$P=\frac{TP}{FP+TP}$$

(4) $$R=\frac{TP}{FN+TP}$$

(5) $$AP=\int_{0}^{1}P(R)dR$$

(6) $$F_{1}=2\times \frac{P\times R}{P+R}$$

where TP represents the number of sperm detected and correctly predicted, TN represents the number of non-sperm detected, FP represents the number of sperm detected but incorrectly predicted, FN represents the number of sperm missed, and AP represents the area of a certain category under the P−R curve. In addition, frames per second (fps) was used to measure the detection speed of the model. The larger the fps, the faster the detection speed of the model.

### 3.2. YOLOv5s Ablation Study

We designed an ablation experiment to verify the effectiveness of the DWConv and SA modules. Each group of experiments used the same parameters and was trained in the same network environment. Table 2 shows the influence of different optimization methods on the detection performance of YOLOv5s on the test dataset. It can be seen from the experimental results that only DWConv is used to replace part of the convolution structure of the YOLOv5s backbone network, and the sperm detection performance is not affected, while the number of network parameters is reduced. The detection speed also increases with the reduction of parameters, from 52.5 fps to 55.8 fps, increasing the recall rates by 1.8 percentage points. With the addition of only the SA attention module, although the optimization method increases the number of parameters and the calculation amount, the accuracy of the sperm detection is increased by 2.1 percentage points, and the recall rate is increased by 2.2 percentage points. The two improvements alone are not significant. However, when the two improvements are added to the original model, the sperm detection accuracy and recall rate are 5.7% and 8.6% higher, respectively, than that of the original YOLOv5s model, and the AP is increased by 6.9% from 80.9% to 87.5%. This shows that the improvement of the two methods has greatly improved the accuracy of the sperm detection.

**Figure 4:**
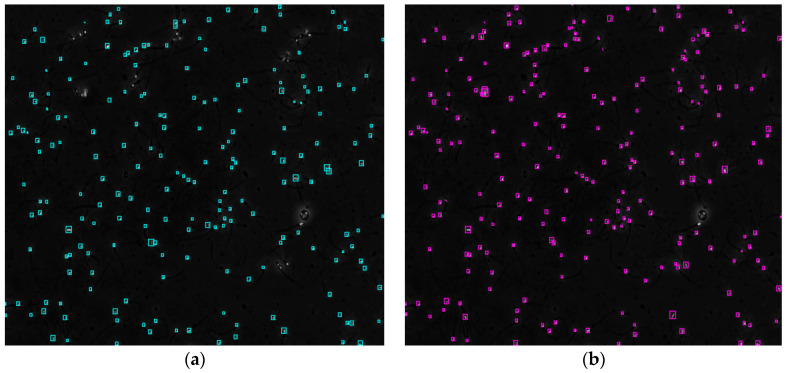
Comparison of model improvement before and after. (**a**) YOLOv5s sperm detection; (**b**) YOLOv5s-SA sperm detection.

Figure 4 shows the comparison of the sperm detection results before and after the network model optimization. It can be seen from Figure 4a that the original YOLOv5s model is prone to missed detection and false detection. Figure 4b shows that the optimized YOLOv5s-SA model significantly improves the defects of the original YOLOv5s sperm detection, and the introduction of the SA attention mechanism can make the model more fine-grained to express multi-scale features, pay more attention to small targets and improve the accuracy of the network sperm detection.

### 3.3. Evaluation of Sperm Detection Methods

The YOLOv5s-SA algorithm is compared with traditional image processing, YOLOv3, YOLOv3-spp, YOLOv5s and YOLOv5m algorithms to objectively evaluate the superiority of YOLOv5s-SA. The same training parameters and datasets are used for training, and the same test set is used for comparison experiments after training. The comparison of evaluation indicators of the different algorithms is shown in Table 3. It can be seen that the detection performance of the traditional image processing methods is not as good as that based on deep learning. YOLOv3 achieved an 83.0 AP on the validation dataset, but the accuracy rate decreased on the test dataset. The YOLOv3 algorithm has only an AP of 69.4% on the test dataset, which is not enough to detect sperm targets effectively, and has poor robustness for the detection of densely distributed sperm. Compared with the YOLOv3-spp algorithm, the YOLOv5s and YOLOv5m algorithms increased by 2.5% and 4.3%, respectively, on the validation dataset. The average precision of the proposed method on the validation dataset is 93.1%, and the average precision on the test dataset is 87.5%, which is 1.9% and 1.9% higher, respectively, than YOLOv5m. In general, the YOLOv5 algorithm can basically recognize the majority of sperm, but it will seriously miss detection in dense and occluded conditions. The detection performance of the YOLOv5s-SA algorithm is improved compared with the YOLOv5 algorithm, and it can maintain a high average precision when the number of parameters is low. In addition, for the case of dense distribution, the number of missed detections is significantly reduced, and the detection effect of the proposed algorithm is the best, and all sperm are basically completely identified.

Speed is a highly critical indicator of sperm target detection. The traditional digital image processing method has reached 1.59 fps, though it only uses the CPU. However, it provides low detection accuracy. Obviously, the deep learning method improves the detection speed (dozens of times as much as the traditional image processing method). In addition, the accuracy of the application of the deep learning method has also been greatly improved. We used a feature extraction network with a more lightweight structure to further improve the speed of sperm detection. Although the SA attention module was introduced in the multi-scale feature fusion process, the detection speed did not decrease significantly. Compared with traditional image processing methods and YOLO series algorithms, the YOLOv5s-SA algorithm can obtain a higher detection accuracy with fewer parameters, and the speed is about three times faster than YOLOv3.

### 3.4. Partial Occlusion Handling

**Figure 5:**
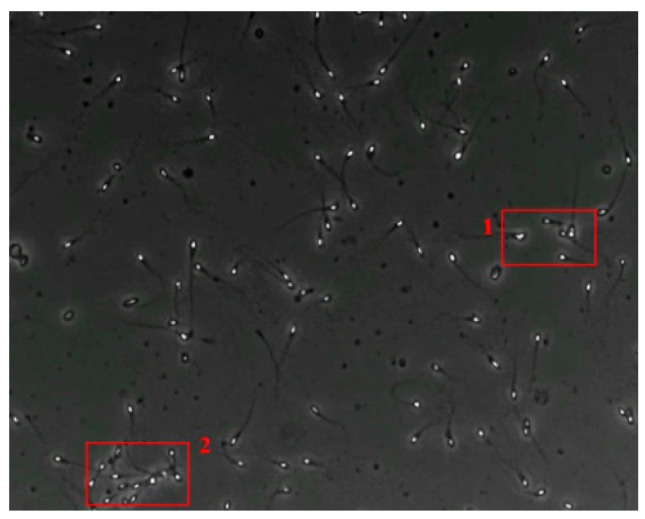
The partial occlusion analysis. (Numbers 1 and 2 represent cases 1 and 2 in Table 4, respectively.)

The small size and fast movement of sperm make it easy to obstruct each other and to create obstructions between sperm moving forward and between sperm and impurities. However, partial occlusion is one of the major challenges resulting from the limited accuracy of sperm detection. Compared with other methods, the improved YOLOv5s-SA model proposed performs well in dealing with partial occlusions. Figure 5 shows a frame of video in the test dataset, and Table 4 shows the comparison details.

In the first state, YOLOv3-spp and YOLOv5s failed to detect partially occluded sperm cells. Our improved YOLOv5s-SA and traditional image processing methods were able to completely detect partially occluded sperm. However, traditional image processing methods failed to detect some obvious sperm correctly. In the second state, YOLOv5s can detect partially occluded sperm. At the same time, it detected some sperm-like impurities as sperm cells, leading to a false detection. Other methods still misjudge or fail to detect. It can be seen that YOLOv5s-SA can accurately detect sperm and has a better effect in handling non-sperm objects, although in a complex background.

### 3.5. Failure Case Analysis

In short, the YOLOv5s-SA model we proposed can detect sperm better than other methods. However, we still encountered some error detection issues. Table 5 shows some detection failures highlighted with arrows.

Taken together, the reasons for these failures are: (1) A large number of fragments in semen are more or less similar to sperm cells (Case 1). (2) The sperm head or neck is attached to the edge of the video frame, making it look like an artifact (Case 2). (3) Sperm is almost completely occluded (case 3).

## 4. Conclusions

As the YOLO algorithm still has insufficient representation ability and low accuracy when applied to small-target detection tasks, we adopted the improved YOLOv5s algorithm for small-target detection of sperm, which is robust for partially blocked sperm, a large number of non-sperm objects and low contrast. DWConv is used to replace partial convolution in the backbone network, which reduces network redundant computation and improves the convergence speed of the model. By integrating the SA attention mechanism into the neck network, the weight relationship between the channels of different scale feature maps was optimized, which alleviated the semantic information attenuation in the process of multi-scale feature fusion and enhanced the multi-scale sperm detection performance. The experimental results show that the proposed YOLOv5s-SA model has significantly improved the detection accuracy of sperm targets, and the detection speed is better than other mainstream methods on the basis of maintaining the lightweight calculation cost. In the future, we hope to extend the proposed method to detect sperm cells in other animals, such as pigs, cows and fish. We believe that the YOLOv5s-SA algorithm has strong generalization ability and robustness, and should also perform well in similar situations of small-size target detection.

## Figures and Tables

**Table 1:** Dataset proportions.

Samples	Video1∼Video6		Video7	Video8	Video9	Video10
Number of frames	Training	Validation	Testing			
	480	120	30	30	30	30

**Table 2:** Ablation experiments. (● represents using the optimization method, ○ represents not using the optimization method).

Model	DWConv	SA	P (%)	R (%)	AP (%)	Params (M)	FPS
YOLOv5s	○	○	77.4	79.6	80.6	7.19	52.5
YOLOv5s + DWConv	●	○	76.3	81.1	80.6	7.06	55.8
YOLOv5s + SA	○	●	76.8	81.5	81.3	7.27	53.0
YOLOv5s + DWConv + SA	●	●	83.1	88.2	87.5	7.14	57.3

**Table 3:** Comparative experimental results.

Model	Validation Set AP (%)	Test Set				Params (M)	FPS
		P (%)	R (%)	AP (%)	F_1_		
Digital image processing	79.2	55.7	55.9	60.1	55.8	n/a	1.59
YOLOv3	83.0	67.3	66.0	69.4	66.6	61.85	19.6
YOLOv3-spp	86.9	75.0	71.4	72.3	73.2	63.53	17.2
YOLOv5s	89.4	77.4	79.6	80.6	78.5	7.19	52.5
YOLOv5m	91.2	82.2	85.4	85.6	83.8	21.07	46.1
YOLOv5s-SA	93.1	83.1	88.2	87.5	85.6	7.14	57.3

**Table 4:** Partial occlusion handling comparison.

Case	Conventional Image Processing	YOLOv3-spp	YOLOv5s	YOLOv5s-SA
1	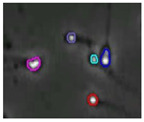	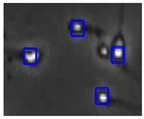	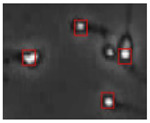	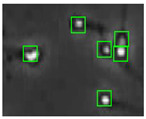
2	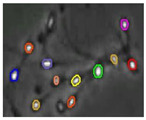	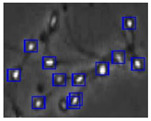	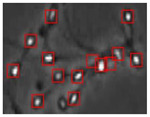	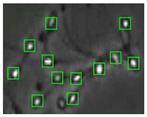

**Table 5:** Some detection failures.

Case	Original Frame	Detection Result
1. Failure example from the validation dataset (false positive)	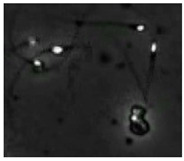	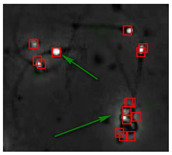
2. Failure example from the test dataset (false positive)	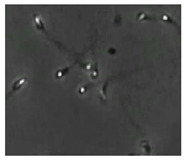	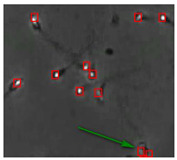
3. Failure example from the test dataset (false negative)	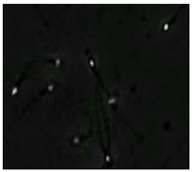	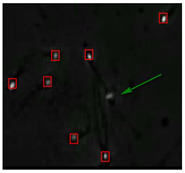

## Data Availability

Not applicable.
